# “What makes life good?” Developing a culturally grounded quality of life measure for Alaska Native college students

**DOI:** 10.3402/ijch.v72i0.21180

**Published:** 2013-08-05

**Authors:** Dinghy Kristine B. Sharma, Ellen D. S. Lopez, Deborah Mekiana, Alaina Ctibor, Charlene Church

**Affiliations:** 1 Center for Alaska Native Health Research, University of Alaska Fairbanks, Fairbanks, AK, USA; 2 Department of Rural Student Services, University of Alaska Fairbanks, Fairbanks, AK, USA

**Keywords:** quality of life, Alaska Native, college students, community-based participatory research

## Abstract

**Background:**

Alaska Native (AN) college students experience higher attrition rates than their non-Native peers. Understanding the factors that contribute to quality of life (“what makes life good”) for AN students will help inform supportive programs that are congruent with their culture and college life experiences.

**Objectives:**

Co-develop a conceptual model and a measure of quality of life (QOL) that reflects the experiences of AN college students.

**Methods:**

Six focus groups were conducted with 26 AN college students. Within a community–academic partnership, interactive data collection activities, co-analysis workgroup sessions and an interactive findings forum ensured a participant-driven research process.

**Findings:**

Students identified and operationally defined eight QOL domains (values, culture and traditions, spirituality, relationships, basic needs, health, learning and leisure). The metaphor of a tree visually illustrates how the domains values, culture and traditions and spirituality form the roots to the other domains that appear to branch out as students navigate the dual worldviews of Native and Western ways of living.

**Conclusions:**

The eight QOL domains and their items identified during focus groups were integrated into a visual model and an objective QOL measure. The hope is to provide a useful tool for developing and evaluating university-based programs and services aimed toward promoting a positive QOL and academic success for AN students.

The long-standing educational and health disparities experienced by Native peoples are well documented (1). At the University of Alaska Fairbanks (UAF), Alaska Native (AN) college students experience higher attrition rates than their non-Native peers (2,3). This is in part due to the challenges that the majority of these students face while navigating a college environment, that is, culturally and physically different from their home communities. To the best of our knowledge, there is no published literature that explains how AN college students conceptualize quality of life (QOL). Nor are there QOL measures that are culturally specific, practical and reflective of their life experiences. This has posed a significant challenge to university-based service providers striving to address the needs of AN students.

It is within this context that we adhered to principles of community-based participatory research (CBPR) (4,5) to engage AN college students in: (a) exploring the factors that contribute to their QOL; (b) conceptualizing a model of their QOL; and (c) developing a culturally grounded QOL measure that can be used to inform the development, implementation and evaluation of support endeavours aimed toward promoting AN college students’ retention and academic success.

## Background

Quality of life is an important indicator when creating and evaluating health and social service programs for diverse populations (6). Despite its widespread use as an assessment or outcome measure, there is little consensus as to how QOL is understood and defined (7). One encompassing conceptualization defines QOL as “a broad range of human experiences related to one's overall well-being” (8, p. 60). In general, QOL as a construct pertains to various dimensions of human experiences that encompass multiple domains in a person's life (9). These include objective life conditions and subjective personal appraisals (10) ranging from life's basic necessities to one's sense of satisfaction and fulfilment (11).

Mukherjee (12) describes two prevailing perspectives in QOL research: One focuses on indicators determined by society as to what people need; the other pertains to what people desire to improve their lives. For example, subjective well-being refers to one's evaluation of personal happiness, and the value he or she places on life at any given time (11). QOL in this sense is closely tied to a person's perception of “meaning” and is often considered to be central to the human condition (13). What is meaningful varies from person to person and depends on the individual's current life conditions, expectations and experiences (14). One of the challenges to measuring QOL is its subjectivity and continual evolution per the individual's adaptation and coping function, level of expectation, and sense of optimism, self-concept, and self-control (15).

### AN students and their QOL

At the UAF, AN students experience a higher attrition rate when compared with their non-Native peers (16). Located in the second largest urban centre in Alaska, UAF offers 167 degrees and 33 certificates within 127 disciplines. Enrolment during fall 2010 was just over 11,000 students, of whom 77% were from Alaska, with 21% self-identifying as AN (14). At the core of UAF's mission to “promote academic excellence, student success and lifelong learning” (5) is the university's quest to serve all Alaskans, with an emphasis on providing educational opportunities to AN and rural students (14,17). As an open admission institution, the objective is not only enrolment of rural and AN students but also successful degree achievement. Yet, according to the UAF Department of Planning, Analysis and Institutional Research, compared to a 72% retention rate among non-Native students, AN students had a retention rate of only 57.4%. As such, of the 674 AN students who registered for the 2009 fall semester, almost half (287) did not return in fall 2010.

Several university-based programs aim to assist rural students as they transition from high school to college (16). Yet studies highlight the need to focus on the college-based experiences of students already enrolled in school. Such experiences have been found to play a pivotal role in a student's ability to thrive and persist in the university setting (18). Nevertheless, little is known about the factors that impact AN students’ decision or ability to continue their college pursuits (18).

Corring and Cook (19) recommend that QOL measurement development integrate individuals’ own definitions of QOL as opposed to relying on QOL measures determined solely from the researchers’ perspectives. Following this advice, our community-academic partnership collaborated with AN college students in exploring their perceptions of QOL within the context of their culture and value systems, and in relation to their goals, expectations and life situations (6). Here we report findings from a focus group study conducted with AN college students that generated a conceptual model and an objective, culturally grounded measure of QOL.

## Methods

### The what makes life good partnership

The CBPR approach has been recommended for working with Native communities (16,17). Ensuring participation in research is essential given the history of mistrust toward research that persists among AN people wherein individuals and communities have been demoralized by research that has posed little benefit or even detriment to their well-being (17).

The What Makes Life Good Project was conducted within a partnership between the Department of Rural Student Services (RSS) and the Center for Alaska Native Health Research (CANHR). Although both are UAF entities, our partnership reflects a community–academic collaboration where CANHR serves as the academic partner, and RSS serves as a prominent overseer to the university's community of rural, and primarily AN, students.

CAHNR is a Center for Biomedical Research Excellence funded by the National Institutes of Health. Since 1999, CANHR's mission has been to work within community-academic partnerships to conduct research and interventions focused on preventing and reducing health disparities experienced by AN people (20,21). RSS was established in 1969 in response to students’ demand for a program that specifically serves AN and rural students. RSS's mission is to meet students’ needs and help them maintain academic and personal balance as they pursue their college education (22).

Within this partnership we conducted a two-phase mixed methods project. Phase I (the focus of this report) involved a formative, qualitative study that employed focus groups, co-analysis workgroup sessions and an interactive findings forum. The objectives were to accomplish the following: (a) explore how AN college students conceptualize and define QOL; (b) determine QOL dimensions and factors that impact its achievement; (c) develop a conceptual model of QOL; and (d) create an objective QOL measure for use with AN college students. Phase 2 involved administering the new QOL measure to AN students to assess their college QOL and to conduct tests assessing the measure's validity and reliability. Here we report on findings that emerged from Phase 1. The study was approved (with exempt status) by the UAF Institutional Review Board.

### Participant eligibility and recruitment

Students were eligible to participate in a focus group if they were currently enrolled at UAF, 18 years or older, AN (self-identified), willing and able to share their views in English, and agreeable to audio-recording focus group sessions. Students were recruited using a combination of purposive and snowball sampling strategies (23). Specifically, recruitment involved word of mouth and disseminating colourful flyers that detailed the purpose of the study and eligibility criteria. To show appreciation, participants received a $30 gift card and a meal.

### Data collection

Focus groups were selected because of their potential to promote interaction and idea exchange among participants and their capacity for exploring local norms, expectations, values and beliefs (24). Data collection processes were based on a review of the QOL and AN literature and with continuous input from RSS staff members and students. The focus group protocol was pilot-tested with RSS staff members who provided constructive feedback.

Six focus groups were conducted over a six-month period (January–June 2011). Each group comprised three to six students and lasted approximately two hours. To foster representation and a comfortable environment, focus group composition included three mixed gender groups, one male-only group, one female-only group and one “non-traditional learners” group (students who were older, enrolled part-time, working full time, married and/or with children). All focus groups were conducted on the UAF campus in the RSS department's gathering room. Sessions were facilitated by the first author with assistance from other research team members. Importantly, at least two AN students (who participated in a focus group) volunteered during subsequent focus groups, helping participants complete consent forms and demographic questionnaires, and taking notes.

### Focus group process

Prior to conducting the focus groups, participants read and signed an informed consent form and completed a brief demographic questionnaire. With permission, focus groups were digitally audio-tape recorded. To facilitate engagement and student-driven data collection and analysis, focus groups comprised interactive, round-robin strategies, including a “word association warm-up” and an “all on the wall activity.”

#### Word association warm-up

During an initial project planning meeting, RSS staff and students explained that AN people often greet each other by asking: “How are you? Good or not so good?” In response, our partnership chose to focus on the research question “what makes life good?” while also maintaining the academic theme “quality of life.” In this way, students could work with a more tangible and culturally congruent term (having a good life) while also providing understanding to the more abstract, though, ubiquitous term (QOL) that is often used by researchers and service organizations.

**Fig. 1:**
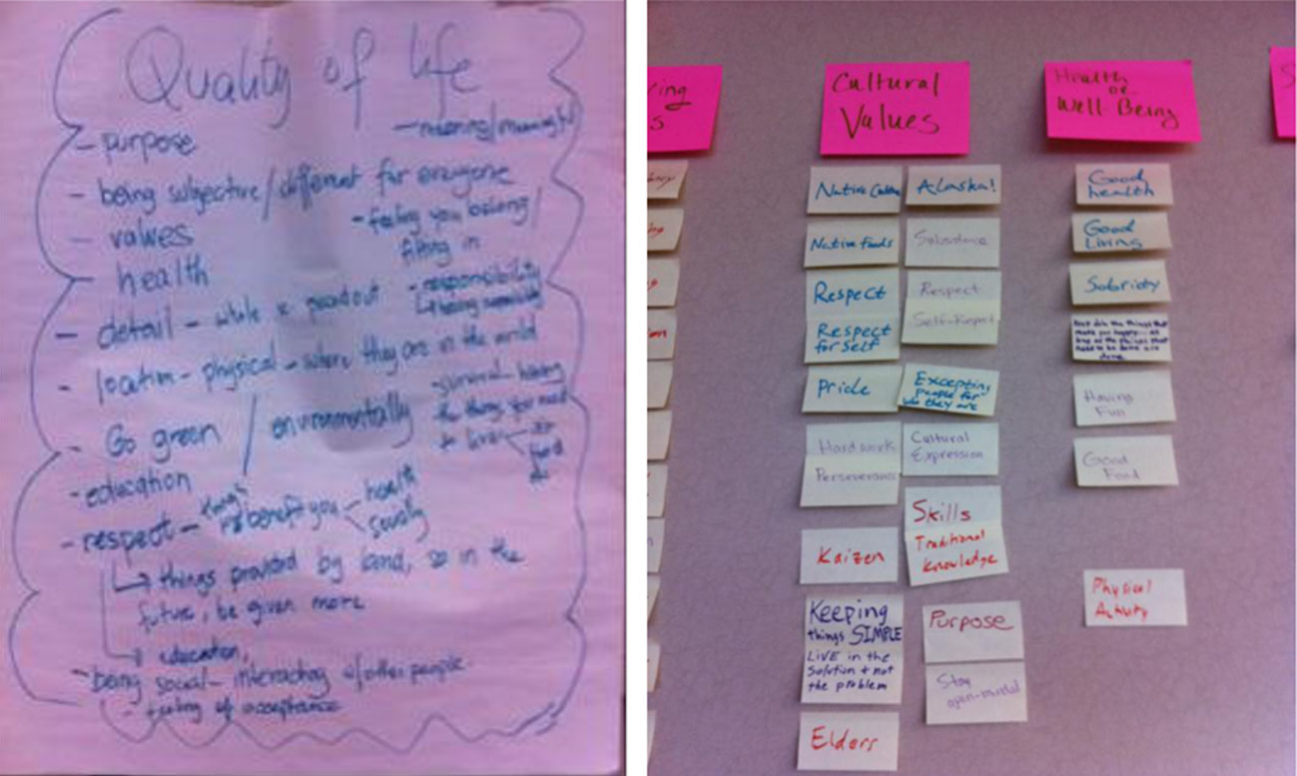
Warm-up word association and all on the wall.

To help prepare students to consider the factors that contribute to their QOL, we opened each focus group with a word-association warm-up that paired QOL with “what makes your life good?” On a sheet of flip chart paper, students were asked to share words or phrases they associated with “life.” On another flip chart sheet, they were asked to do the same for “quality.” They then were asked to share terms they associated with “quality of life.” Figure 1 (left) shows the terms participants in one of the focus groups associated with QOL.

#### All on the wall activity

Each student was then provided with a pad of sticky notes. On each sticky (as many as needed) participants were asked to “write one of the things you consider to be important to your QOL as an AN college student.” One by one, students shared what they wrote on each of their sticky notes and then posted them on a blank wall. For example, one student's list included eating Native foods, financial aid and having friends at school. When the sticky notes for all participants were shared and posted, the students worked together to group them into thematic domains. Then they named each domain to reflect the meaning it represented to their QOL. Examples from the list of 35 themes that emerged across the six focus groups included: cultural values, career, hobbies, health and safety, religion, education and family. Figure 1 (right) provides an example of how participants in one focus group classified items under the theme, cultural values.

### Data management, analysis and member checking

The demographic questionnaires were coded and analyzed using descriptive statistics through IBM SPSS computer software. All focus groups were audio-recorded, but not transcribed; recordings were used to ensure that all items and themes were captured and accurately defined. Beyond the thematic analysis that occurred during the focus groups, data were further interpreted and synthesized during co-analysis workgroup sessions. From June–September 2011, researchers and at least two AN students worked together to accomplish the following: (a) distil the 35 domains that emerged across the six focus groups into non-overlapping domains; (b) develop a conceptual model to illustrate QOL based on the domains; and (c) format the QOL domains and items into a new QOL measure.

Member checking occurred in October 2011 during an Interactive Findings Forum where RSS staff, focus group participants and researchers gathered to hear and discuss findings. During the forum, participants reviewed a draft version of the QOL measure and provided feedback for improvement.

## Findings

### Respondents

A total of 26 AN students took part in focus groups. Participants included 18 females and eight males. Ages ranged from 18–26 ($\bar{x}$=22, M=21). Most participants (n=23) were from rural Alaska communities and identified with the following AN ethnicities, Yup'ik (n=11), Athabascan (n=5), Inupiaq (n=4), Aleut (N=2), Cup'ik (N=1), Haida (N=1), Inupiaq-Tlingkit (n=1) and Inupiaq-Yup'ik (n=1). Primarily, students reported pursuing four-year degrees from a variety of disciplines, including science, rural development, liberal arts, AN studies, business administration and education. Most (n=23) described themselves as single. Five students reported having at least one child. All except two students reported coming to college from a rural Alaska community.

### QOL domains and items

Ultimately we identified and operationalized eight QOL domains: culture and traditions, spirituality, values, relationships, learning, basic needs, leisure and health. Table I provides the operational definition for each domain and examples of items (students’ sticky notes) grouped under them.

**Table 1:** Eight QOL domains with their operational definitions, and examples of items

Domain	Operational definition	Examples of items (per sticky notes)
Culture and traditions	Practices, expressions and activities pertaining to AN culture	Engaging in subsistence activities (fishing, hunting, trapping, berry-picking) Participating in cultural gatherings (dancing, singing, drumming, storytelling)
Spirituality	Practice, expressions, and activities pertaining to faith and/or religion	Attending religious events and/or gatherings Having faith in God or another higher power
Values	Traditional and Western belief systems and principles that guide thinking and behaviors	Respecting Elders and parents Respecting the land Taking responsibility to fulfil obligations Being honest with self and others
Relationships	Social connections maintained with others—at home or at college (in person, phone Internet)	Talking to family members Having good relationship with advisors Having network of friends (in person and online)
Learning	Services, resources, and activities that promote academic learning	Receiving a college education Having quality and supportive professors Receiving scholarships
Basic needs	Essentials for survival during college	Having shelter, food, money Feeling safe in the community
Leisure	Activities that are fun, entertaining, and promote stress-management and relaxation	Hanging out with friends Having fun/cool things to do Participating in traditional activities Engaging in outdoor sports and activities
Health	Behaviors and activities that promote and maintain health	Getting exercise, sleep, health foods Being cigarette/tobacco free Practicing safe sex; having access to condoms Have access to health care

**Fig. 2:**
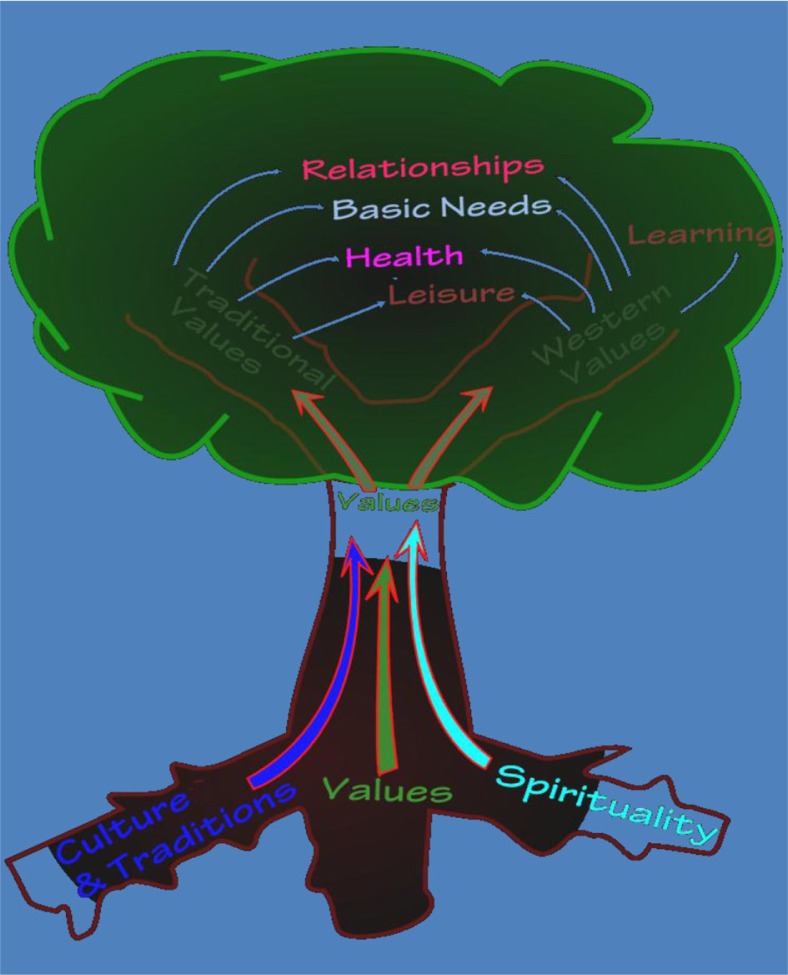
Conceptual model of the QOL domains of AN students.

From discussions and analysis of these domains and the QOL items, a conceptual model of AN students’ QOL emerged (see Fig. 2). In examining the domains and the items they represent, a metaphor of a tree developed where the domains culture and traditions, spirituality and values form the foundation (the tree's roots and trunk) to students’ QOL. Overlapping and repeated items identified by students across the six focus groups were related to these three domains and so formed their foundational salience to college students’ QOL. This conceptualization is consistent with existing literature highlighting the significance of cultural values, tradition and spirituality to Native well-being (24–26). Moving up the tree, the values domain branches off in two directions illustrating how students must navigate and strike a balance between traditional (such as a strong sense of community, subsistence lifestyle) and Western (such as competition, individualism) ways of life that are at times in conflict. Further, branching off from values are the domains of relationships, learning, basic needs, leisure and health. Interestingly, the learning domain (comprising items reflective of Western education) branches from the Western values boughs of the tree, while the leisure, relationships, basic needs and health domains included items reflective of both traditional and Western values. The visual conceptual model of AN students’ QOL proved useful in concretely illustrating and translating research findings to the RSS community (staff and students) and other stakeholders.

## QOL measure

Our partnership developed a QOL measure for use with AN college students. The measure comprised demographic questions and all of the QOL items grouped (during analyses) under each of the eight domains. The items were formatted as five-level Likert scale items so students could rate each according to importance, frequency and satisfaction.

Using the QOL measure will be useful to RSS and other stakeholders as it can quantify how specific items and domains impact students’ QOL in terms of their necessity, salience and current fulfilment. Hence, the information generated through this measure will be helpful in the process of prioritizing what support services can be enhanced or developed, and how they may be evaluated.

## Interactive findings forum

To provide structured feedback about the QOL measure, RSS students and staff who participated in the interactive findings forum completed a form that instructed them to read through each of the measure's items and provide feedback about wording, relevance and cultural sensitivity. Participants noted questions that should be omitted or added and were asked to comment on the format and length of the measure. For example, despite noting that the survey was long (more than 100 questions), students were reluctant to omit any items because they all seemed relevant to their college QOL. Instead, they suggested keeping all of the questions and seeing how the number of items might be reduced (through factor analysis) after pilot testing the measure with a larger group of AN students. Forum participants also suggested moving the demographic questions to the beginning of the survey because AN students were accustomed to “introducing” themselves when initiating interactions with others. They also determined that the survey should be made available both online and via paper/pencil.

## Next steps

During Phase II of the study, we pilot-tested the QOL measure with 111 AN students and subjected the data to principal component analysis and item response theory analysis. This resulted in a significant reduction of items; therefore, a new version of the QOL measure was developed, with improved reliability and validity. During a future interactive findings forum, survey results and the current measure will be presented to the RSS community again for further discussion and feedback.

## Summary and conclusion

We discussed findings and products from the first phase of a collaborative, mixed methods research process aimed at gaining perspectives from AN college students of “what makes life good?” with the goal of developing a culturally-grounded QOL measure. Our findings confirm that QOL for AN college students is, indeed, a complex and highly subjective construct as it pertains to their unique life situations (14). Because our goal was to develop a practical and relevant QOL measure for use with AN college students, it was critical to gain perspectives and interpret findings with AN students themselves. Adhering to the principles of community-based participatory research (CBPR) helped ensure that all aspects of the research (design, data collection, analysis and measurement development) were conducted within a true partnership, and that findings were interpreted through the insight of local knowledge. The result is a QOL measure that is grounded within the student-determined and defined domains of QOL (culture and traditions, spirituality, values, basic needs, learning, relationships, health and leisure). We likewise co-developed a conceptual model that visually illustrates how culture, spirituality and values form the foundation of QOL for students as they navigate the duality of traditional and Western values in order to achieve social, educational, physical and mental well-being.

The findings (items, domains and conceptual model) from this study concur with those reported from other studies investigating perceptions of well-being by indigenous people. For example, for Yup'ik AN people (in southwest Alaska), discussions of health and wellness emphasized the significance of traditional values and connections to community and nature to healing and sustaining a sense of well-being (24–26). Similarly, Inuit people in Nunavut (in arctic Canada) conceptualized well-being and happiness as including strong family and kinship support and a connection to the land and cultural traditions (25). These related findings signify that what makes life good for Native people is closely intertwined with their traditional practices. For AN college students, QOL appears to be rooted in cultural, spiritual and traditional values. Yet achieving a good life at college requires the blending of traditional and Western ways toward learning, relaxing, connecting with others, maintaining health and meeting basic needs.

Inherent to qualitative research, findings from this study have limited generalizability beyond the community of AN students associated with the Rural Student Services. Further, although efforts were made to represent gender, and traditional and non-traditional students, focus group participants were primarily younger, female, traditional students. As such, caution should be used when applying findings to other student populations and settings.

There is growing evidence that interventions developed for indigenous people will be successful if they are based within their cultural beliefs and practices, and if they are developed through community engagement (25). The AN college QOL measure that resulted from our CANHR-RSS partnership is based within the reality of life for AN students. As such it holds promise as a resource to those who strive to serve our AN students and as a tool for student advocacy. Data provided by this new measure can inform genuinely responsive efforts aimed toward promoting a positive QOL and academic success for AN college students.
